# The prevalence and burden of systemic lupus erythematosus in a medicare population: retrospective analysis of medicare claims

**DOI:** 10.1186/s12962-015-0034-z

**Published:** 2015-05-06

**Authors:** Cindy Garris, Manan Shah, Eileen Farrelly

**Affiliations:** GlaxoSmithKline, Research Triangle Park, Durham County, NC USA; Bristol-Myers Squibb, Tampa, FL USA; Xcenda, Palm Harbor, FL USA

**Keywords:** SLE, Lupus, Medicare, Disability, Burden of illness, Cost, Healthcare resource utilization

## Abstract

**Background:**

Systemic lupus erythematosus (SLE) is a chronic autoimmune disorder which can affect multiple organs of the body, requiring ongoing disease management and healthcare resource utilization. The economic impact of SLE has not been evaluated in a Medicare population to date. This study was conducted to assess the prevalence of SLE and its burden in terms of healthcare resource utilization and costs in a US Medicare population.

**Methods:**

This was a retrospective observational study using Medicare medical claims data (5% random sample) for the period spanning 2003 to 2007. SLE patients were identified by having ≥2 medical claims with a primary or secondary diagnosis of ICD-9 code 710.0X. The earliest quarter of SLE diagnosis was defined as the index quarter. Prevalence of SLE, the proportion of SLE cases on disability benefits, and the contribution of SLE to new disability cases were evaluated. Healthcare resource utilization and direct medical costs (2008 US dollars) over 12 months were compared between a cohort of patients with SLE and a cohort without SLE matched on key demographics. Differences in outcomes between cohorts were assessed using McNemar’s test for dichotomous variables and paired t-tests for continuous variables.

**Results:**

A total of 13,348 patients with SLE were identified. The prevalence of SLE was approximately 3 per 1000 Medicare beneficiaries. After matching, the sample consisted of 6,707 SLE and 13,414 non-SLE patients. On average, the SLE cohort compared with the non-SLE cohort had 2.4 times more physician visits, 2.7 times more hospitalizations, 2.2 times more outpatient visits, and 2.1 times more emergency room visits. A medical cost surplus of approximately $10,229 per patient per year in the SLE cohort relative to the non-SLE cohort was driven largely by inpatient hospitalization costs (p < 0.001).

**Conclusions:**

SLE prevalence was 3 per 1,000 Medicare patients. Patients with SLE consumed significantly more health care resources with significantly greater costs compared with those without SLE. Added costs were largely attributable to inpatient hospitalizations. The Medicare population is an important target for efforts to improve SLE disease management and reduce costs.

## Background

Systemic lupus erythematosus (SLE) is a chronic autoimmune inflammatory disorder that causes significant morbidity and mortality [1]. SLE has an estimated incidence of 5 per 100,000 persons and a prevalence of 100 per 100,000 persons in the United States [2-5]. A relapsing-remitting condition in which periods of mild disease activity alternate with flares of increased disease activity, SLE is manifested mainly in women, but also in children and men, with clinical and pathologic manifestations involving almost all bodily organs [1,6,7]. Symptoms can include fatigue, joint pain and sensitivity, malar rash on the face, and skin photosensitivity. Complications can include kidney damage, including kidney failure, cognitive and memory impairment, neuropsychiatric symptoms, blood disorders and cardiovascular disease [8-10]. Although SLE is currently incurable, survival rates and longevity have increased in recent decades with improvements in diagnosis and therapy [1,5,11]. Management of SLE focuses on reducing disease activity and preventing or reducing disease flares [7], which impair quality of life and cause disability with associated reductions in productivity and loss of employment [12-20].

The economic impact of SLE has been evaluated in managed care or Medicaid populations [21-25], but not assessed to date in a Medicare population. In 2010, Medicare provided healthcare coverage for approximately 47.5 million Americans (39.6 million ages 65 and older, and 7.9 million disabled) and enrollment is projected to reach 78 million by 2030 [26]. Generally, individuals ages 65 years and older who have a ≥5-year status as legal residents of the United States are eligible for Medicare as are disabled people younger than 65 years who receive Social Security Disability Insurance benefits and people who receive dialysis for end-stage renal disease or need a kidney transplant [26]. The increasing longevity of patients with SLE [1,5,11], frequent renal involvement including the need for dialysis and kidney transplant [27-29], and the association of SLE with disability [14,15,18,19], potentially make Medicare a major source of healthcare coverage for SLE patients and could render the cost of SLE in the Medicare population substantial. The present study was conducted to assess the prevalence of SLE and its burden in terms of healthcare resource utilization and costs in a US Medicare population, for both the overall Medicare population and for those patients <65 years of age receiving disability benefits through Medicare.

## Methods

This retrospective analysis (GHO-09-1623) assessed the prevalence of SLE, the proportion of SLE cases on disability benefits, the contribution of SLE to new disability cases each year, and healthcare resource utilization and costs in a Medicare population from 2003–2007.

### Database and sample

This analysis was conducted using a 5% random sample of Medicare’s medical claims database obtained from the Centers for Medicare and Medicaid Services (CMS). This database is a nationally representative sample of patients seeking medical care through Medicare providers and can be extrapolated to the entire Medicare population. It contains all inpatient fee-for-service claims (Part A) and outpatient fee-for-service claims (Part B) for beneficiaries. Prescription data (Part D) was not available for this study. Actual dates of service are not available in the database, therefore the quarter in which the service was rendered is utilized.

Inclusion criteria for the target SLE cohort were patients having 2 or more medical claims with a primary or secondary diagnosis of SLE (International Classification of Diseases, 9^th^ edition (ICD-9) code 710.0x) during the identification period (first quarter of 2003 through the fourth quarter of 2006). The index quarter was defined as the earliest quarter of SLE diagnosis for the SLE cohort. For the non-SLE control group, inclusion criteria required that patients never had SLE during the study period. The index quarter for non-SLE patients was defined as the first quarter of Medicare enrollment for patients 65 years and older or the first quarter with a qualifying disability for patients under the age of 65 years. Patients were excluded from the non-SLE control group if they were <65 years of age with end stage renal disease (ESRD). Patient-level data were de-identified and thus exempt from institutional board review and ethics committee approval. The study was conducted in accordance with the principles of the Health Insurance Portability and Accountability Act (HIPAA).

### Prevalence of SLE

The prevalence of SLE during each study year 2003–2007 was calculated as the number of SLE cases in a given calendar year divided by the number of Medicare beneficiaries who were continuously eligible during the same calendar year. The date of the first SLE claim was used in the prevalence calculations.

### SLE patients receiving disability benefits

The proportion of patients with SLE receiving disability benefits in a given year from 2003–2007 was calculated as the number of SLE cases with qualifying disability divided by the total number of SLE cases. Medicare beneficiaries (including SLE patients) were deemed to receive disability based on the entitlement code (value of 1=’disability insurance benefits’ or 3=’disability insurance benefits and ESRD’) listed on claims. The proportion of new disability cases attributed to SLE was calculated as the number of new SLE cases with qualifying disability in a given year from 2003–2007 divided by the number of new disability cases in that year. The date of the first SLE claim was used in the disability analyses.

### Healthcare resource utilization and costs

Healthcare resource utilization and healthcare costs were compared between cohorts with and without SLE matched 1:2 on the basis of age (±2 years), gender, race, region, and index quarter (±2). To be eligible for the matched sample, patients had to have a first SLE diagnosis that occurred from the first quarter of 2003 through the fourth quarter of 2006 (index quarter) and had to be continuously eligible for medical services during the 2 quarters before and the 1 year after the index quarter.

Outcomes were evaluated for 1 year after the index quarter and included all-cause healthcare utilization and costs (inflated to 2008 US dollars) for inpatient hospitalizations, emergency room visits, physician visits, and outpatient visits. Outpatient drug costs were not determined because these records are not included in the Medicare Part A or Part B datasets. Healthcare utilization and costs were summarized both for the matched sample as a whole and for the subset of the matched sample on disability benefits. Healthcare costs were also summarized in the matched sample as a whole as a function of race and gender. Re-hospitalization rates and average length of stay were also calculated.

### Statistical analysis

Inferential statistics was used to describe and quantify inter-cohort differences in baseline characteristics before the cohorts were matched. After matching, baseline characteristics were compared between cohorts using paired t-tests or Wilcoxon signed rank tests (continuous variables) and McNemar’s test (categorical variables). SLE prevalence, the proportion of SLE cases on disability benefits, and the contribution of SLE to new disability cases were summarized with descriptive statistics. Outcomes were compared statistically between cohorts using paired t-tests for continuous variables and McNemar’s test for categorical variables. Outcome comparisons were conducted for the sample as a whole and stratified by race and gender. Differences between cohorts were considered statistically significant at p < 0.001. SAS software was used for statistical analysis (© SAS Institute Inc, Cary, NC).

## Results

### Prevalence of SLE

**Figure 1:**
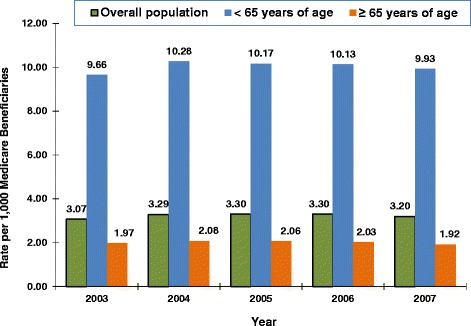
Prevalence of SLE in a Medicare population: 2003–2007.

The number of Medicare beneficiaries for the study period (5% random sample) was 2,719,310 of whom 13,348 had 2 or more claims for SLE. The prevalence of SLE in the overall Medicare population from 2003–2007 (Figure 1) remained relatively stable at approximately 3 per 1000 Medicare beneficiaries (range 3.07-3.30). The SLE prevalence among Medicare beneficiaries < 65 years of age receiving disability was approximately 10 per 1000, while the prevalence for the ≥ 65 Medicare population was approximately 2 per 1000.

### SLE patients receiving disability benefits

The number of new disability cases per year in the 5% random sample of the Medicare claims database remained relatively stable (range 35,597-37,704) from 2003–2007. The proportion of new disability cases attributed to SLE ranged between 1.4-2.0%. Of the patients in the overall SLE cohort, the proportion receiving disability benefits increased from 45.0% in 2003 to 49.7% in 2007.

### Patient sample and demographics

A total of 13,348 patients met criteria for inclusion in the SLE cohort and 2,705,962 patients met criteria for the non-SLE cohort. Patient characteristics prior to matching are shown in Table 1. Matching identified a match for half of the SLE cohort (N = 6,707). These patients were matched 1:2 to 13,414 control patients without SLE. Patient characteristics were similar across the matched cohorts (Table 2). Mean age was 61 years and 86% were female. Half of the patients in each cohort were receiving disability benefits.

**Table 1:** **Patient characteristics pre-matching**

	**Medicare disabled age <65 years**		**Medicare age ≥65 years**	
**Characteristics**	**SLE cohort**	**Non-SLE cohort**	**SLE cohort**	**Non-SLE cohort**
	**N = 5,555**	**N = 455,950**	**N = 7,793**	**N = 2,250,012**
Mean age, years	49	50	74	74
Female, %	87*	46*	83**	57**
Race, %				
White	62.6*	73.3*	84.3	85.5
Black	28.3*	18.4*	10.1	8.2
Other	9.2	8.3	4.4	6.3
Region, %				
Northeast	15.9*	18.4*	19.2	19.9
Midwest	18.9*	21.4*	19.7**	23.0**
South	46.3*	39.9*	42.3**	35.0**
West	16.8*	17.6*	17.7**	20.1**
Other	2.1	2.8	1.2	2.1

*P < 0.05 between Medicare Disabled age <65 cohorts.

**P < 0.05 between Medicare age ≥65 cohorts.

**Table 2:** **Patient characteristics post-matching**

**Characteristic**	**SLE cohort**	**Non-SLE cohort-matched controls**
	**N = 6707**	**N = 13,414**
Disability benefits, %	49.9	49.9
Mean age, years	61	61
Female, %	86.0	86.0
Race, %		
White	73.1	73.1
Black	20.3	20.3
Other	6.6	6.6
Region, %		
Northeast	16.4	16.4
Midwest	19.5	19.5
South	45.5	45.5
West	16.9	16.9
Other	1.7	1.7
Index year, %		
2003	81.1	79.6
2004	7.6	7.5
2005	6.1	6.3
2006	5.2	6.7

No significant differences between groups.

### Healthcare utilization

**Figure 2:**
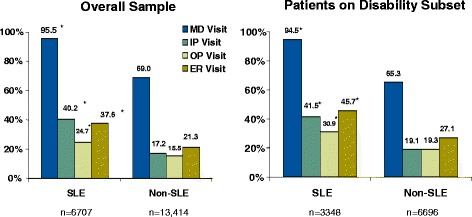
Annual Healthcare Resource Utilization – proportion of patients. *p < 0.0001 versus non-SLE group. MD = physician visits; IP = inpatient visits; OP = outpatient visits; ER = emergency room visits.

**Figure 3:**
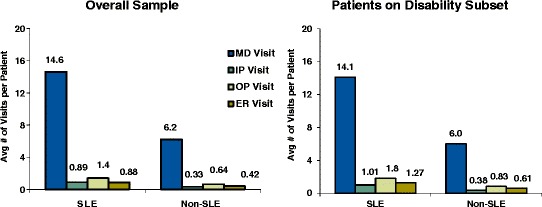
Annual Healthcare Resource Utilization – average number of visits. *p < 0.001 for each comparison of SLE vs. Non-SLE in the overall sample and disability subset.

In the matched sample overall, the percentage of patients in the SLE cohort who utilized all categories of healthcare, including physician visits, in-patient visits, outpatient visits, and emergency room visits, was significantly larger than the percentage utilized by the non-SLE cohort (Figure 2). The biggest difference was seen in hospitalizations (40.2% in SLE cohort vs. 17.2% in the non-SLE cohort, p < 0.001). Likewise, the average annual number of uses of each of these categories of healthcare was significantly higher in the SLE cohort than the non-SLE cohort (Figure 3). The same pattern of significant differences in utilization (proportion of patients and average number of visits) between the SLE and non-SLE cohorts was observed in the subset of the matched sample on disability benefits (Figures 2, 3), and when the total sample was stratified by race and gender (data not shown).

In post-hoc analyses of patients with at least one hospitalization, the average length of hospital stay was 6.4 days for both the SLE and non-SLE cohorts. Re-hospitalization rates were numerically higher (not tested for significance) in the SLE cohort (53%) compared with the non-SLE cohort (43%).

### Healthcare costs

**Figure 4:**
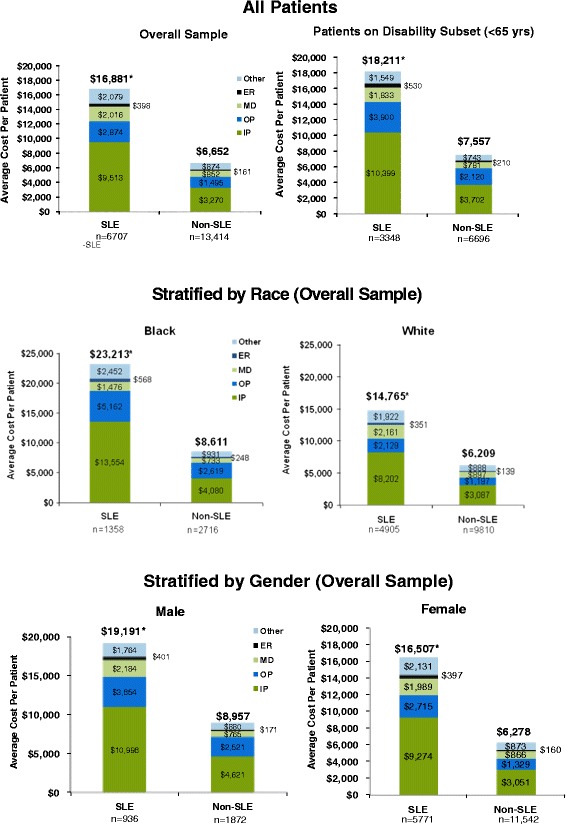
Average Annual Healthcare Costs per Patient. ER = emergency room; MD = physician visit; OP = outpatient hospital visit; IP = inpatient hospital stay; Other = other medical costs. *P < 0.0001 versus non-SLE group.

Patients with SLE incurred significantly greater average annual healthcare costs than matched controls without SLE (Figure 4). The cost surplus of approximately $10,330 (2008 US dollars) in the SLE cohort relative to the non-SLE cohort was driven primarily by inpatient hospital costs, which were approximately 2.7 times greater in patients with SLE. Costs for other categories of healthcare use, including outpatient visits, physician visits, and emergency room visits were at least 2 times greater in the SLE cohort compared with the non-SLE cohort. The same pattern of significant differences in cost between the SLE and non-SLE cohorts was observed in the subset of the matched sample on disability benefits, and when the total sample was stratified by race and gender (Figure 4). The magnitude of the difference in healthcare costs between the SLE cohort and the non-SLE cohort was greater for blacks than for whites, but was similar between males and females.

## Discussion

Although several cost of illness studies have been conducted in patients with SLE in managed care or Medicaid populations, this is the first study to evaluate the prevalence, healthcare resource utilization and cost of SLE in a sample of Medicare beneficiaries. The prevalence of SLE in this large US Medicare population was found to be approximately 3 per 1000 across the 5-year study period spanning 2003 to 2007. This prevalence rate exceeds the approximate 1 per 1000 persons estimate obtained from several studies conducted in various settings during the past 3 decades [2-5]. The higher prevalence of SLE overall in this Medicare sample is driven by the increased prevalence of SLE in the disabled cohort (approximately 10 per 1000) compared with the SLE patients >65 years of age (approximately 2 per 1000).

The findings that approximately half of SLE patients in this study were on disability benefits and that approximately 2% of new disability cases were attributed to SLE are consistent with previous reports linking SLE to functional impairment, poor quality of life, reduced productivity, and unemployment [12-20]. In a review of 12 studies (including patients from US and Europe) on employment and disability in SLE, the prevalence of inability to work or cessation of work ranged from 15% to 51% across studies and 20% to 32% of SLE patients received disability benefits [14]. While our analysis found that approximately half of SLE patients received disability benefits under Medicare, the large variance in percentage of patients receiving disability benefits in the review paper is likely attributable to the sources of data and differing definitions of disability benefits since patients from countries other than the US were included. Patients with SLE are actually less successful in attaining federal disability assistance than patients with other diseases because medical records may not accurately reflect functional limitations, and SLE symptoms that contribute to work disability like fatigue, pain, and neurocognitive dysfunction are difficult to assess and quantify [14].

In the current study, patients with SLE in the Medicare population were significantly more likely to use healthcare resources than the matched control group without SLE, and the higher rate of healthcare resource utilization translated into significantly higher healthcare costs. On average, patients in the SLE cohort had at least two times more physician visits, hospitalizations, outpatient visits, and emergency room visits when compared to the non-SLE cohort —excess utilization that translated into $10,229 greater annual healthcare costs per patient. The annual per-patient healthcare cost of SLE in this study ($16,881 in 2008 USD) was similar to or lower than the annual costs found in other studies of a managed care or Medicaid population (approximately $16,000-$30,000) [12,22-25]. While this study’s annual health care cost figure does not include outpatient drug costs given that outpatient drug costs are not included in Medicare Part A or Part B datasets, previous studies demonstrate that medication costs contribute a relatively small proportion (6-23%) of direct healthcare costs for SLE patients in studies of economic burden of SLE in managed care [12,24,25].

The difference in healthcare costs between the SLE cohort and the non-SLE cohort was similar between the matched sample as a whole and the subset of the matched sample on disability benefits. The difference in costs between the SLE cohort and the non-SLE cohort was also similar between males and females in the matched sample as a whole but was magnified in blacks compared with whites. Factors such as (a) greater disease severity attributable to genetic and non-genetic (environmental and socioeconomic) factors in blacks and other minority groups [5,30] and (b) that severe flares of disease activity have been shown to incur higher costs [31] may explain the latter finding, and warrants further elucidation.

Inpatient costs were the primary drivers of the difference in healthcare costs between the SLE cohort and the non-SLE cohort. Reasons for hospitalization among these Medicare recipients with SLE were not determined in this study. These reasons may be of interest, however, as a study by Ward et al. [32] found that being on Medicare as opposed to having other types of health insurance was a risk factor for avoidable hospitalization in patients with SLE. Increasing age and lower socioeconomic status were also associated with avoidable hospitalization in the study. Ward suggested that as Medicare can be obtained by patients receiving Social Security Disability Insurance, Medicare might constitute a marker for more severe illness than observed with other types of healthcare insurance. The reasons for hospitalization of patients with SLE merit systematic investigation in the context of the large contribution of hospitalizations to the total medical cost of SLE.

One limitation to interpretation of study results is the possible misidentification of Medicare beneficiaries with SLE. The identification of SLE patients was based on diagnosis codes where the potential for coding errors exists. The potential bias was minimized by requiring patients to have at least 2 separate medical claims with a primary or secondary diagnosis of SLE. Second, prescription medication data (i.e. Medicare Part D) was not available for inclusion in the study, which would increase the total economic burden of SLE. Third, clinical and disease-specific parameters that could impact study outcomes are not readily available in claims data. Despite the lack of clinical parameters, claims data are very useful in evaluating real-world resource utilization and cost patterns.

## Conclusions

This study quantifies the prevalence and economic burden of SLE in a large Medicare population. The prevalence of SLE in this Medicare study sample (3 per 1,000 overall and 10 per 1,000 for disabled beneficiaries), was higher than the national prevalence estimate of 1 per 1000 people. More than 40% of all SLE cases in the Medicare sample (before matching) were patients <65 years of age receiving disability benefits. SLE is associated with high healthcare resource utilization and costs, on average 2 times higher utilization and $10,000 greater annual costs than non-SLE patients, with inpatient hospitalization as the largest cost driver. Thus, the Medicare population is an important patient segment to direct efforts to improve disease management strategies and reduce costs of SLE.

## Abbreviations

CMS: Centers for medicare and medicaid services
ESRD: End stage renal disease
ICD-9: International classification of diseases, 9^th^ edition
MD: Physician office visit
ER: Emergency room
IP: Inpatient hospitalization
OP: Outpatient hospitalization
SLE: Systemic lupus erythematosus
